# A glutamatergic biomarker panel enables differentiating Grade 4 gliomas/astrocytomas from brain metastases

**DOI:** 10.3389/fonc.2024.1335401

**Published:** 2024-05-21

**Authors:** Falko Lange, Richard Gade, Anne Einsle, Katrin Porath, Gesine Reichart, Claudia Maletzki, Björn Schneider, Christian Henker, Daniel Dubinski, Michael Linnebacher, Rüdiger Köhling, Thomas M. Freiman, Timo Kirschstein

**Affiliations:** ^1^ Oscar-Langendorff-Institute of Physiology, University Medical Center Rostock, Rostock, Germany; ^2^ Center for Transdisciplinary Neurosciences Rostock, University of Rostock, Rostock, Germany; ^3^ Hematology, Oncology, Palliative Medicine, University Medical Center Rostock, Rostock, Germany; ^4^ Institute of Pathology, University Medical Center Rostock, Rostock, Germany; ^5^ Department of Neurosurgery, University Medical Center Rostock, Rostock, Germany; ^6^ Molecular Oncology and Immunotherapy, Clinic of General Surgery, University Medical Center Rostock, Rostock, Germany

**Keywords:** glioblastoma, brain metastasis, glutamate, glutamate receptors, biomarker, epilepsy

## Abstract

**Background:**

The differentiation of high-grade glioma and brain tumors of an extracranial origin is eminent for the decision on subsequent treatment regimens. While in high-grade glioma, a surgical resection of the tumor mass is a fundamental part of current standard regimens, in brain metastasis, the burden of the primary tumor must be considered. However, without a cancer history, the differentiation remains challenging in the imaging. Hence, biopsies are common that may help to identify the tumor origin. An additional tool to support the differentiation may be of great help. For this purpose, we aimed to identify a biomarker panel based on the expression analysis of a small sample of tissue to support the pathological analysis of surgery resection specimens. Given that an aberrant glutamate signaling was identified to drive glioblastoma progression, we focused on glutamate receptors and key players of glutamate homeostasis.

**Methods:**

Based on surgically resected samples from 55 brain tumors, the expression of ionotropic and metabotropic glutamate receptors and key players of glutamate homeostasis were analyzed by RT-PCR. Subsequently, a receiver operating characteristic (ROC) analysis was performed to identify genes whose expression levels may be associated with either glioblastoma or brain metastasis.

**Results:**

Out of a total of 29 glutamatergic genes analyzed, nine genes presented a significantly different expression level between high-grade gliomas and brain metastases. Of those, seven were identified as potential biomarker candidates including genes encoding for AMPA receptors *GRIA1*, *GRIA2*, kainate receptors *GRIK1* and *GRIK4*, metabotropic receptor *GRM3*, transaminase *BCAT1* and the glutamine synthetase (encoded by *GLUL*). Overall, the biomarker panel achieved an accuracy of 88% (95% CI: 87.1, 90.8) in predicting the tumor entity. Gene expression data, however, could not discriminate between patients with seizures from those without.

**Conclusion:**

We have identified a panel of seven genes whose expression may serve as a biomarker panel to discriminate glioblastomas and brain metastases at the molecular level. After further validation, our biomarker signatures could be of great use in the decision making on subsequent treatment regimens after diagnosis.

## 1 Introduction

High grade glioma (CNS WHO grade 4) and brain metastases represent the most frequent tumors in the CNS (1, 2). Yet, the therapies of both diseases differ fundamentally. In case of a glioblastoma, treatment aims to total bulk resection in combination with subsequent radio-chemotherapy (3), whereas in the case of brain metastases, the primary tumor must be taken into account (4). Differentiating both tumor entities in the radiological imaging may be challenging (5). Once a primary extracranial malignancy is known, a tumor bulk in the MRI is more likely to be a brain metastasis. A multifocal appearance is also indicative of an extracranial origin of the tumor. In the case of single bulk, however, diagnosis may be ambiguous. Several MRI-based studies presented approaches to address this demand, that may in the future possibly become useful as additional tools to predict the tumor entity (6–8). Currently, the gold standard is still a histopathologic assessment. Tissue biopsies are common when MRI does not lead to an unequivocal diagnosis. Hence, additional biomarkers easy to obtain would be of great interest to distinguish glioblastoma from metastasis.

In glioblastoma, various pathophysiological processes were identified that drive the progression of the disease (9), including aberrant glutamatergic mechanisms (10–12). In patients suffering from glioma, the extracellular glutamate levels surrounding the tumor mass were identified as elevated (13, 14). High levels of glutamate contribute to hyperexcitability of tumor-surrounding neurons, epileptic seizures, and in the end, may favor tumor bulk expansion by excitotoxicity (15, 16). Since survival of patients suffering from glioblastoma is limited to approximately 15 months, maintaining the quality of life by preventing seizures is one of the main goals. To achieve seizure-free conditions, anticonvulsants targeting glutamate signaling are frequently in use (17, 18). On the molecular level, glutamate is released from the tumor cells in exchange for cystine via solute carrier family 7 member 11 (SLC7A11; xCT), an antiporter that was found to be upregulated in glioma (19). Cystine is an essential precursor for glutathione synthesis to address oxidative stress. Furthermore, the Na^+^-dependent uptake of glutamate via solute carrier family 1 member 2 (SLC1A2; EAAT2) is impaired by downregulation or mislocalization of the transporter (20–22). In isocitrate dehydrogenase 1 (IDH1) wildtype glioblastoma, branched chain amino acid transaminase 1 (BCAT1) is also often upregulated and may contribute to a glutamatergic phenotype (23, 24). In addition to glutamate shuttling and metabolism of glioma cells, ionotropic and metabotropic glutamate receptors were identified to contribute to the tumor progression (11). The glioblastoma cells express α-amino-3-hydroxy-5-methyl-4-isoxazolepropionic acid (AMPA) receptors to a varying extent (25, 26), and exposure to AMPA receptors inhibitor perampanel resulted in antitumoral effects (27, 28). In neuro-gliomal synapses, transmission via AMPA receptors favored the glioma progression (29–31). With respect to metabotropic glutamate receptors, group II receptors (mGluR2 and mGluR3) in particular contributed to cancer growth (32–34).

The impact of an aberrant glutamate signaling in non-neuronal cancers is highly variable and less well studied (summarized in Ref (35). and Ref (36).). While glutamate receptors and transporters were found to be altered in most malignancies such as lung carcinoma (37–39) and pancreatic adenocarcinoma (40, 41), the overall glutamatergic phenotypes are less pronounced than in tumors of neural or glial origin. Therefore, aiming for identifying a mechanistically driven biomarker panel to distinguish glioblastoma and brain metastasis at the pathomolecular level, we investigated the expression of glutamate receptors and key players of glutamate homeostasis in human tumor tissue samples. Since aberrant glutamate signaling may contribute to tumor-associated epilepsy, we further asked whether gene expression patterns might differ between glioblastoma patients suffering from seizures and those without reported epilepsy.

## 2 Materials and methods

### 2.1 Patients and tumor samples

Tumor tissue samples were obtained from patients treated at the Department of Neurosurgery of the Rostock University Medical Center, Germany from 2011 to 2023. Inclusion criteria were diagnosed CNS WHO Grade 4 glioma (IDH1-wildtype) and CNS WHO grade 4 astrocytoma (IDH1-mutant) or brain tumors with extracranial origin (42). In this study, IDH1-wildtype and IDH1-mutant were merged in one high-grade primary brain tumor group (referred to as high-grade glioma or HGG). In our study, tissues from a total of 55 individuals (29 male and 26 female), with informed written patient consent (ethics registration IDs: A45-2007, A2018-0167, A2019-0187) were included. All procedures involving patients were approved by local Ethics Committee (University Medical Center Rostock). Patients that were initially diagnosed as glioblastoma prior surgical resection, but in the subsequent histopathological assessment rated as low-grade glioma, were excluded from the study. The clinical data were obtained from the Department of Neurosurgery, the Department of Neurology, and the Institute of Pathology. An overview of the two patient cohorts (including data on sex, age, tumor localization, origin of the primary tumor for MET and information on molecular status (IDH mutation, MGMT promoter methylation) for HGG) is presented in **Supplementary Table 1** and **Supplementary Table 2** respectively. Process of diagnosis of the tumor entity was conducted as described in the German guidelines on glioma, that is based on WHO classification and suggestions of the ciMPACT-NOW consortium (42). The presence of epileptic seizures was clinically documented via patient history and/or was diagnosed by additional EEG analysis.

### 2.2 RNA isolation and cDNA synthesis

To extract RNA from snap-frozen surgical samples, tissues of the size of approx. 3x3x3 mm^3^ were pestled employing a vibration mill (MM 400 Mixer Mill, Retsch, Haan, Germany) and subjected to TRIzol reagent (Invitrogen, Carlsbad, CA, USA). RNA isolation was performed according to the manufacturer’s instructions. Afterwards, any traces of genomic DNA were removed employing DNA-free™ DNA Removal Kit (Invitrogen). For cDNA synthesis, all reagents were from Promega Corporation (Madison, WI, USA). For a total volume of 25 µl, 1 µg RNA was reverse-transcribed into cDNA by means of Moloney Murine Leukemia Virus Reverse Transcriptase, RNase H Minus, Point Mutant (200 U) and RNasin Plus RNase inhibitor (25 U) in the presence of random hexamers (0.25 μg) and dNTP Mix (0.4 mM each). Initially, the random hexamers and the RNA were incubated for 5 min at 70°C. The following sequence was 10 min at 20°C, 50 min at 40°C followed by 15 min at 70°C. All synthesized cDNAs were quantified and stored at -80°C until further usage.

### 2.3 Quantitative RT-PCR

Relative quantification of target cDNA levels by real-time PCR was performed in a qTOWER^3^ detection system (Analytik Jena AG, Jena, Germany). Therefore, AceQ qPCR SYBR Green Master Mix (Absource Diagnostics, Munich, Germany) and human gene-specific primers (TIB Molbiol, Berlin, Germany; **Supplementary Table 3**) were used. *GAPDH* (glyceraldehyde-3-phosphate dehydrogenase) and *TBP* (TATA-box binding protein) served as house-keeping gene controls. All data were analyzed for both housekeeping genes. In the current manuscript, the data based on *TBP* are presented, since TBP expression was found to be more robust in glioblastoma (43). Primer sequences (see **Supplementary Table 3**) for ionotropic and metabotropic glutamate receptors were obtained from Ref (25). and genes associated with glutamate homeostasis were from Lange et al., 2019 (27). PCR conditions were 95°C for 5 min, followed by 40 cycles of 10 s at 95°C/30 s at 60°C. To further address the quality of the primers used in our study, melting curves at the end of each RT-PCR were recorded (15 s; 0.1°C-steps; see **Supplementary Figure 1** and **Supplementary 2** for sample melting curves for each gene). Real-time PCR products were subjected to gel electrophoresis (2% agarose). Here, for each set of primers only one PCR product was determined (**Supplementary Figure 3**). For each biological sample the relative expression of each mRNA (based on technical duplicates) compared to the housekeeping gene *TBP* was calculated according to the equation $\Delta Ct=Ct_{target}-Ct_{TBP}$ . The relative amount of target mRNA was expressed as $10^{3}\times 2^{-\Delta Ct}$ .

### 2.4 Immunohistochemistry

Analysis of glutamate receptor expression on the protein level was performed on paraffin-embedded tumor tissues. For this purpose, 5-µm-sections were prepared and deparaffinization was done by a standard protocol. For the immunohistochemistry of GluA1 (encoded by GRIA1) and GluA2 (encoded by GRIA2) heat-induced antigen retrieval (10 min cooking time, 0.05% Tween-20 in 10 mM citrate buffer, pH 6.0) was carried out to enhance the immunofluorescent signal. After cooling down for 20 min and 3×10 min washing in PBS, sections were first incubated for 20 min with 0.1% triton-X (in PBS), washed with PBS (2×10 min), and afterwards incubated for 60 min with 10% normal goat serum (NGS; Thermo Fisher Scientific, Waltham, MA, USA). Next, tissue sections were incubated with the primary antibody for anti-GluA1 antibody (Abcam; ab183797; diluted 1:100), anti-GluA2 antibody (Abcam; ab20629; diluted 1:100) or anti-mGluR3 antibody (Thermo Fisher Scientific; MA5-31749; diluted 1:200) respectively at 4°C overnight. Next day, slices were washed 3x10 min with PBS and were exposed to secondary antibodies (Alexa Fluor 488 goat anti-rabbit (Thermo Fisher Scientific; A-11034; diluted 1:400 in PBS/1% NGS) or Cyanine5 (Cy5) goat anti-mouse (Thermo Fisher Scientific; A10524, diluted 1:200 in PBS/1% NGS). Afterwards, the slices were counterstained and mounted with ProLong Gold Antifade Reagent containing 4’,6-diamidino-2-phenylindole (DAPI, Thermo Fisher Scientific, P36931). Fluorescence analysis was performed by using a laser-scanning microscope (Leica DMI 6000, Wetzlar, Germany) and Leica Application Suite (v. 2.0.0.13332) software. At 100x magnification, regions of interest in the tumor sections were placed and mean fluorescent signals of Alexa Fluor 488, Cy5 and DAPI were estimated. The ratio of the secondary antibody signal and DAPI was calculated to estimate the relative glutamate receptor expressions. Protein expressions were compared with relative mRNA expression by calculating the Pearson correlation coefficients.

### 2.5 Statistical analysis

Statistical analyses were performed with IBM SPSS Statistics (Version 27, IBM, Ehningen, Germany). The expression data are presented as box-and-whisker plots. The box represents 25^th^ and 75^th^ percentiles separated by the median, while whiskers show 10^th^ and 90^th^ percentiles. Outliers are marked as circles. The arithmetic mean is illustrated as a red line. Group differences were tested for significance using the nonparametric Mann-Whitney U test. In the main body of the text, the gene expressions were compared as fold difference of the means between high-grade glioma and metastasis. A receiver operating characteristic (ROC) analysis was used to identify genes with high area under the curve (AUC) values (>0.8) that may serve as potential biomarker candidates. To estimate the optimal cut-points, Youden indices were calculated as sum of sensitivity and selectivity. A t-test was used to compare receptor expression in immunochemical experiments. Pearson correlation coefficients were calculated to estimate the effects of age or sex on the occurrence of epilepsy in Grade 4 glioma/astrocytoma and to compare the expression of selected candidates on protein and mRNA level. A significance level of p<0.05 was considered statistically significant.

## 3 Results

### 3.1 Expression of glutamate receptors and genes associated with glutamate homeostasis in brain tumors

The aim of this study was to identify candidate genes whose expression highly differs between high-grade glioma/astrocytoma (henceforth abbreviated as high-grade glioma or “HGG”) and brain tumors with extracranial origin (brain metastasis or “MET”). In our study, we included a total of 55 patients of whom 35 were diagnosed with HGG (40% female) and 20 suffered from brain metastasis (60% female; **Table 1**). The median age at diagnosis was rather comparable between both cohorts; patients with HGG showed a median age of 68 years (29-91 years) and those with MET a median of 58 years (42-75 years). In the study, 33/35 CNS WHO 4 brain cancers were diagnosed as glioblastoma (IDH1-wildtype) and two were classified as IDH1-mutant astrocytoma (**Supplementary Table 1**). As summarized in **Supplementary Table 2**, metastases derived from lung cancer (n=11), breast cancer (n=3), colorectal/rectal cancer (n=3) or were of renal carcinoma, melanoma, and cervical cancer origin (one patient in each case).

**Table 1:** Overview of Grade 4 glioma/astrocytoma and brain metastasis cohorts.

	HGG (n=35)	MET (n=20)
sex	f:14 | m:21	f:12 | m:8
age (y)	68 (29–91)	58 (42–75)
occur. of epilepsy	51.4%	20%

**Figure 1:**
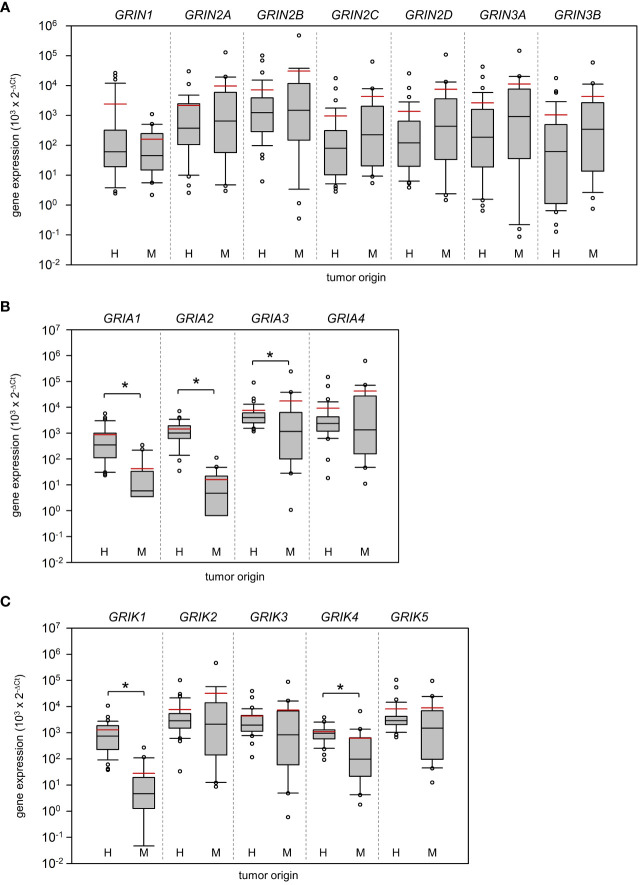
Expression of ionotropic glutamate receptors in Grade 4 gliomas/astrocytomas and brain metastases. RNA was isolated from grade 4 glioma/astrocytoma (H, n=35) and brain metastasis samples (M, n=20), and reverse-transcribed in cDNA as described in the material and methods section. Subsequently, the mRNA expression of **(A)** NMDA receptors, **(B)** AMPA receptors, **(C)** KA receptors and house-keeping control TATA-box binding protein (*TBP*) was assessed by real-time PCR. The box-and-whisker plots represent relative amounts ( $10^{3}\times 2^{-\Delta Ct}$ ) of target mRNA. Median is shown as a black-coloured line and the mean is red; *p<0.05 (Mann-Whitney U test).

**Figure 2:**
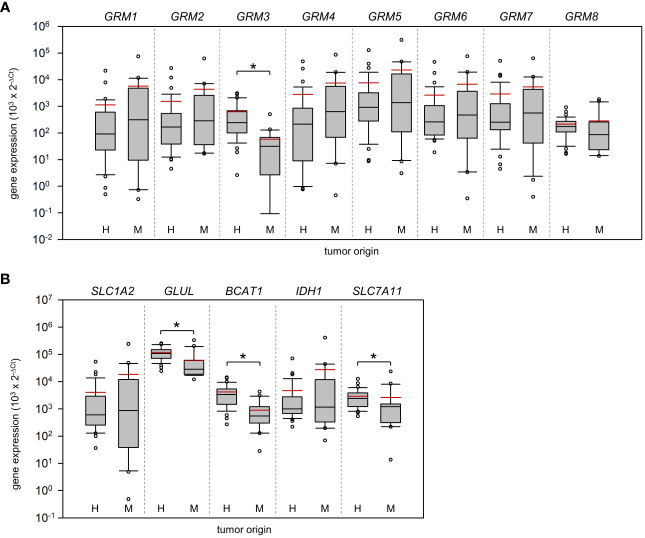
Expression of metabotropic glutamate receptors and key players of glutamate homeostasis in Grade 4 gliomas/astrocytomas and brain metastases. RNA was isolated from grade 4 glioma/astrocytoma (H, n=35) and brain metastasis samples (M, n=20) and reverse-transcribed in cDNA. Subsequently, the mRNA expression of **(A)** metabotropic receptors (*GRM1-8*) and **(B)** key players of glutamate shuttling (*SLC1A2* and *SLC7A11* coding for EAAT2 and xCT antiporter respectively) and metabolism (*IDH1*, *BCAT1*, *GLUL*), and house-keeping control *TBP* was quantified by real-time PCR. The box-and-whisker plots represent relative amounts ( $10^{3}\times 2^{-\Delta Ct}$ ) of target mRNA. Median is shown as a black-coloured line and the mean is red; *p<0.05 (Mann-Whitney U test).

Based on real-time PCR analysis, no significant differences between HGG and MET in the relative expression of N-methyl-D-aspartate (NMDA) receptors were determined (**Figure 1A**; Mann-Whitney U test). In marked contrast, AMPA receptor subunits *GRIA1* (~20-fold difference; p<0.001) and *GRIA2* (~90-fold difference; p<0.001) were found to be higher expressed in HGG than in the MET cohort (**Figure 1B**). Interestingly, the *GRIA3* expression was found to be lower in HGG than in MET (~2.4-fold difference; p=0.01). With respect to kainate receptors, the subunits *GRIK1* (~46-fold difference; p<0.001) and *GRIK4* (~1.8-fold difference; p<0.001) were also higher expressed in HGG than in MET (**Figure 1C**). As shown in **Figure 2A**, *GRM3* is the only metabotropic glutamate receptor gene that showed a differential expression (~11-fold higher expression in HGG; p<0.001) between both patient cohorts (**Figure 2A**).

The last group analyzed were genes that are associated with glutamate shuttling and metabolism. Our analysis revealed that *GLUL* (~17.2-fold difference; p<0.001) a gene encoding for glutamine synthetase, branched chain amino acid transaminase 1 (*BCAT1*; ~4.7-fold difference; p<0.001), and *SLC7A11* (~1.1-fold difference; p= 0.005), encoding the xCT cystine/glutamate transporter, were higher expressed in HGG (**Figure 2B**). Remarkably, no difference in the expression of *EAAT2* (encoded by *SLC1A2*), a Na^+^/glutamate co-transporter previously reported to be downregulated in glioma (21, 22), was determined. Exclusion of astrocytoma samples from the statistical analysis had no impact on the results with respect to significant differences between both cohorts.

**Figure 3:**
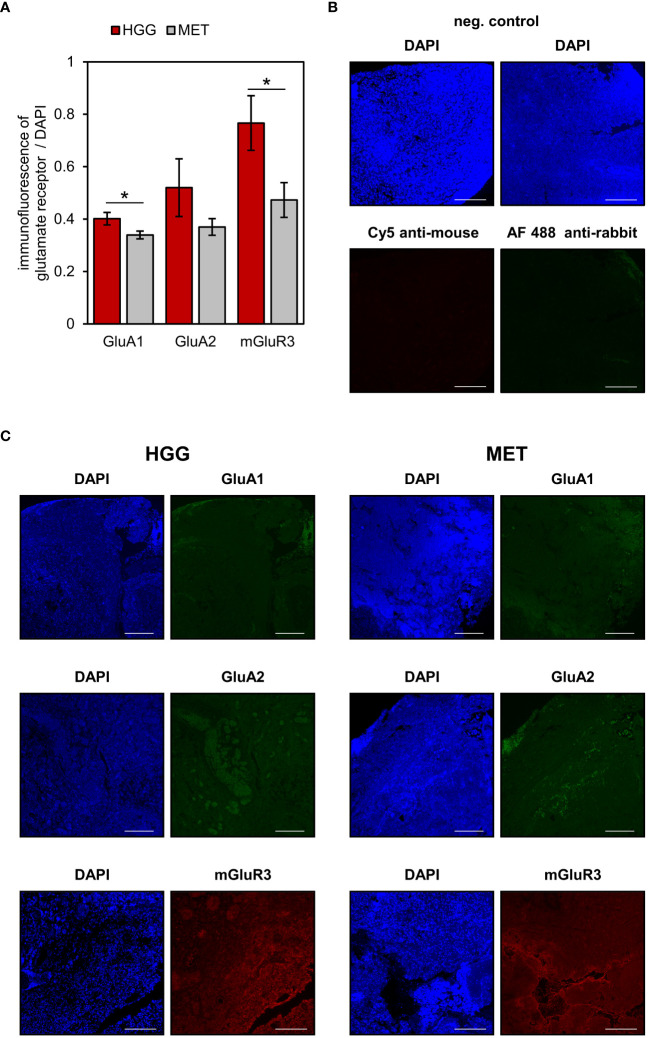
Glutamate receptors expression in human brain tumour slices. AMPA receptor subunits GluA1 and GluA2 (shown in green) and metabotropic receptor mGluR3 (red) were determined in the tumour area. Additionally, nuclei were counterstained with DAPI (blue). **(A)** The fluorescence levels of glutamate receptors and DAPI were used to quantify receptor expression in glioblastoma (n=5) and metastasis (n=5) tissues as described in detail in the material & methods section. Data are presented as mean ± SEM; *p<0.05 (Student’s t-test). **(B)** Negative controls of Alexa Fluor 488 and Cy5-conjugated secondary antibodies. **(C)** Representative images are based on microscopic photographs that were taken at 100× magnification. Bars represent 200 μm.

Three of the differentially expressed glutamate receptors (*GRIA1*, *GRIA2*, *GRM3*) were selected for immunohistological verification of the mRNA expression (**Figure 3**). In a subset of 10 patients (5 samples per tumor cohort), no significant difference was found in the expression of GluA2 between both tumor entities (**Figure 3A**; p=0.227, Student’s t-test). However, in congruence with the gene expression, protein expressions of GluA1 (p=0.038) and mGluR3 (p=0.045) were found to be significantly higher in HGG than in MET. Altogether, correlation between those three candidates on the RNA and protein level failed to reach the significance level (n=10 patients; Pearson correlation coefficient was 0.31; p=0.0951).

### 3.2 No association between the prevalence of seizures and glutamate receptor expression

Next, we asked whether glutamate receptor expression in the tumor tissue was associated with an epileptic phenotype in patients suffering from HGG, as pathophysiological glutamate signaling may contribute to tumor-associated seizures (11, 44). In our study, 51% (n=18) of the patients exhibited an epileptic phenotype (**Table 1**). However, no significant differences in all investigated genes between both HGG cohorts were detected (**Supplementary Figure 4** and **Supplementary Figure 5**). Interestingly, an inverse correlation between the occurrence of seizures and age was determined (n=34 patients; Pearson correlation coefficient was -0.408; p=0.0165). The cohort suffering from epilepsy had a median age of 52 years (29-91 years) and those patients without seizures were 72 years-old (39-80 years). As shown in previous studies (45, 46), sex did not correlate with diagnosed epilepsy (n=34 patients; Pearson correlation coefficient was 0.228; p=0.194).

### 3.3 A ROC analysis elucidated a set of genes to potentially distinguish Grade 4 glioma/astrocytoma and brain metastasis

To further investigate the expression pattern of glutamatergic genes that may help to differentiate HGG and MET on the molecular level, a ROC analysis (sensitivity vs. 1–specificity) was performed. It was hypothesized that among the statistically significant identified nine genes (**Figure 1** and **Figure 2**), potential biomarker candidates could be evaluated. In the ROC analysis, an area under the curve (AUC) with >0.8 was assumed as robust value to distinguish both tumor cohorts.

**Figure 4:**
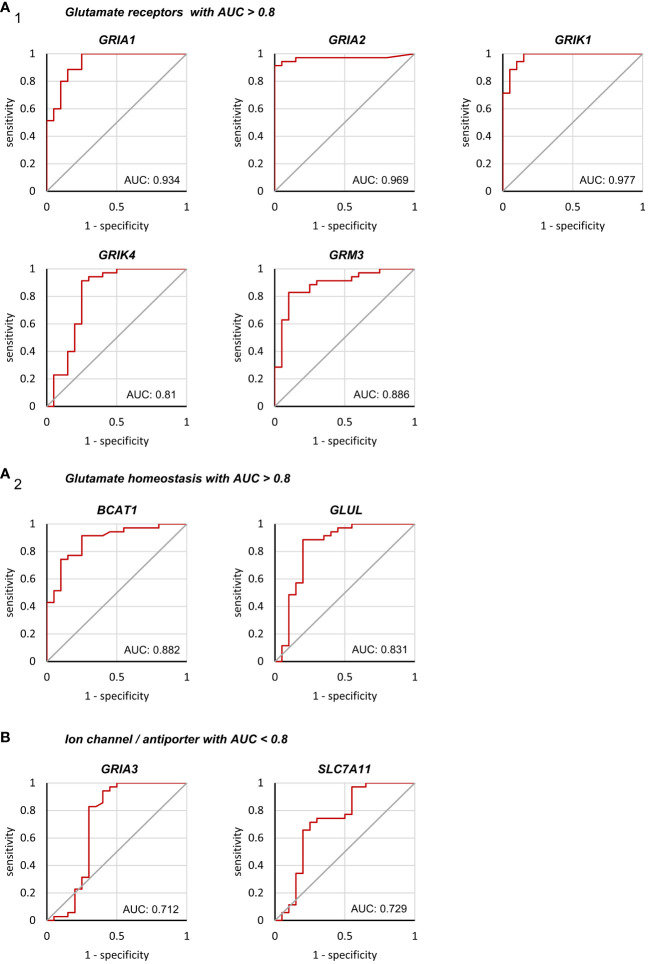
Binary classification of Grade 4 glioma/astrocytoma and brain metastasis by receiver operating characteristic analyses. ROC analyses were performed on a total of 55 tissue samples obtained from surgery (n=35 grade 4 glioma/astrocytoma, n=20 brain metastases). Only genes with an AUC>0.8 are presented in **(A_1_)** encoding for glutamate receptors and **(A_2_)** glutamate homeostasis. **(B)** Both, *GRIA3* and *SLC7A11* failed to reach an AUC>0.8 and were excluded as biomarker candidates (see supplementary 6 and 7 for the remaining genes with AUC<0.8).

In seven of nine genes, an AUC >0.8 was found (**Figure 4**). This included *GRIA1*, *GRIA2*, and *GRIK1* with AUCs even >0.9, and *GRIK4*, *GRM3*, *GLUL* and *BCAT1* with AUC >0.8, while *GRIA3* and *SLC7A11* presented AUCs of 0.712 and 0.729 respectively (**Figure 4B**). All the remaining gene expression patterns presented AUCs <0.7 (**Supplementary Figure 6** and **Supplementary Figure 7**) and were excluded as biomarker candidates.

To estimate the optimal cut-point, Youden indices were calculated for each AUC of the target genes with an AUC >0.8 (**Table 2**). Additionally, the ΔCt values of these cut-points were calculated (**Table 2**). Furthermore, for each biological sample it was determined whether the ΔCt of the Youden indices could be used as a predictor of the tumor entity. On average for all genes, an accuracy of 88% (95% CI: 87.1, 90.8) to predict the correct tumor entity was found (**Table 2**). Hence, a panel of genes was needed to ensure that the correct disease was predicted. In 30 out of 55 tissue samples, the ΔCt values of all seven genes could be used to decide on the correct tumor entity (true positive or true negative). In 14 surgical samples, six of seven were correct. Of the remaining eleven samples, 5/7 (n=4), 4/7 (n=4) and 3/7(n=3) ΔCt values in our model were found to be true positives or true negatives, respectively.

**Table 2:** Youden indices of target gene expressions.

			HGG		MET		HGG+MET
gene	Youden index	ΔCt	correct prediction	% of cohort	correct prediction	% of cohort	accuracy (%)
*GRIA1*	0.736	4.52	31/35	88.6	17/20	85	87.3
*GRIA2*	0.914	2.81	32/35	91.4	20/20	100	94.5
*GRIK1*	0.836	2.85	31/35	88.6	19/20	95	90.9
*GRIK4*	0.664	1.91	32/35	91.4	15/20	75	85.5
*GRM3*	0.729	3.55	29/35	82.9	18/20	90	85.5
*GLUL*	0.686	5.93	31/35	88.6	16/20	80	85.5
*BCAT1*	0.664	0.13	32/35	91.4	15/20	75	85.5
						total accuracy: ~88%	

## 4 Discussion

After determination of a neoplastic mass in the brain, a rapid diagnosis of the tumor entity is crucial for the decision on subsequent treatment regimens. With a history of extracranial malignancy, a brain cancer is often appropriately classified as a metastasis. However, without a cancer history, differentiating high-grade glioma from metastasis remains challenging in the imaging. At least two predictive biomarkers were established in high-grade glioma. Both, *MGMT* promoter methylation and *IDH1* mutations are associated with a better overall survival of patients with primary brain cancers (47). After diagnosis, patients exhibiting a high rate of *MGMT* promoter methylation had a 50% longer median survival when treated with temozolomide (48). However, no molecular fingerprints were proposed to distinguish brain metastasis and glioblastoma. Especially in tumor samples that may be insufficient for precise histopathological examination like biopsy specimens, a set of biomarkers requiring only a small amount of tissue could be a supportive tool.

Our major finding was the identification of a panel of seven genes that may support a differentiation at the pathomolecular level. This set of genes includes all subgroups of receptors and key players of glutamate homeostasis. First, two ionotropic AMPA receptors, GRIA1 and GRIA2 were found to be higher expressed in primary brain tumors. AMPA receptors contributed to migration and survival of glioma cells (49, 50). Currently, AMPA receptors were found to be enriched at the tumor rim in neuro-gliomal synapsis and mediated fast excitatory postsynaptic currents (29, 31). An inhibition of AMPA receptors by perampanel affected proliferation and survival of glioblastoma cells under *in vitro* conditions (27, 51, 52). However, *in vivo* experiments could not confirm the antitumoral effects (53). In accordance with our study, Brocke et al., 2010 found the AMPA receptor subunit GRIA4 to be highly expressed (25). But no difference between metastasis and primary brain cancers were detected for this AMPA receptor subunit. A second group of ionotropic glutamate receptors with potential biomarker candidates are kainate (KA) receptors. The KA receptor subunits encoded by *GRIK1* and *GRIK4* presented a highly differential expression. So far, KA receptor functions in glioblastoma were scarcely investigated. In agreement with our data, juvenile glioblastoma expressed all five subunits of KA receptors (25). KA receptors are primarily expressed in CNS in pre- and post-synaptic membranes, but may also fulfil non-synaptic functions (54). So far, KA receptors were also reported in permanent cell lines including glioblastoma, lung cancer, breast cancer and colon carcinoma (26), but cellular functions remained elusive. Interestingly, NMDA receptors presented no differential expression between both tumor cohorts. Recently, NMDA receptors were identified to be involved in chemoresistance to temozolomide (55) and radiosensitivity (56).

In the group of metabotropic glutamate receptors, mGluR3 (encoded by *GRM3*) was the only receptor subtype with a differential expression, that also was present in a varying protein expression in a subset of the samples. Since, mGluR3 was higher expressed in glioblastoma than most other tumor entities (incl. lung, colon, and breast cancer), it is not an unexpected candidate (57). Our data indicate that the distinction is not primarily due to an overexpression of mGluR3 with respect to other metabotropic glutamate receptors in glioblastoma, but primarily due to a low expression in metastases. In glioblastoma, the expression of mGluR3 is inversely correlated with the survival of patients (57, 58). In preclinical models, an inhibition of mGluR3 *in vitro* revealed antitumoral effects, but failed to prolong survival *in vivo* (57). However, a low expression profile or inhibition of mGluR3 may increase susceptibility to temozolomide (58, 59). In lung cancer, representing the primary origin of more than half of the metastases in our study, mGluR3 was reported to be absent (26) and for this reason may highly affect the overall expression pattern of the metastases cohort.

In the group of glutamate shuttling and metabolism genes, only *BCAT1* and *GLUL* presented an AUC >0.8. The glutamate-synthesizing aminotransferase BCAT1 (produces glutamate from α-ketoglutarate) may overproduce glutamate in tumors and an upregulation of *BCAT1*, that may in part be driven by hypoxic conditions of fast-growing glioblastoma (60), was associated with poor patient survival (61, 62). As a partner in crime, the glutamine synthetase (GS; encoded by *GLUL*) may be upregulated in high-grade glioma (63), whereas other malignancies presented mixed results (64). Glutamine represents a major component in various metabolic cascades of the tumor cells to address energy consumption and demand of newly synthesized nucleotides (65). Furthermore, a high expression of the enzyme is correlated with the prevalence of seizures and in addition a reduced survival (66). Interestingly, we found only a relatively high but not excellent correlation between xCT expression and tumor origin (AUC=0.729). One reason could be the overexpression of xCT in lung carcinoma (38) and colon carcinoma (67) that could have been preserved in the metastases. In marked contrast, Na^+^/glutamate co-transporter EAAT2 described as often downregulated in glioblastoma (22), showed no association between tumor entity and expression at all.

**Figure 5:**
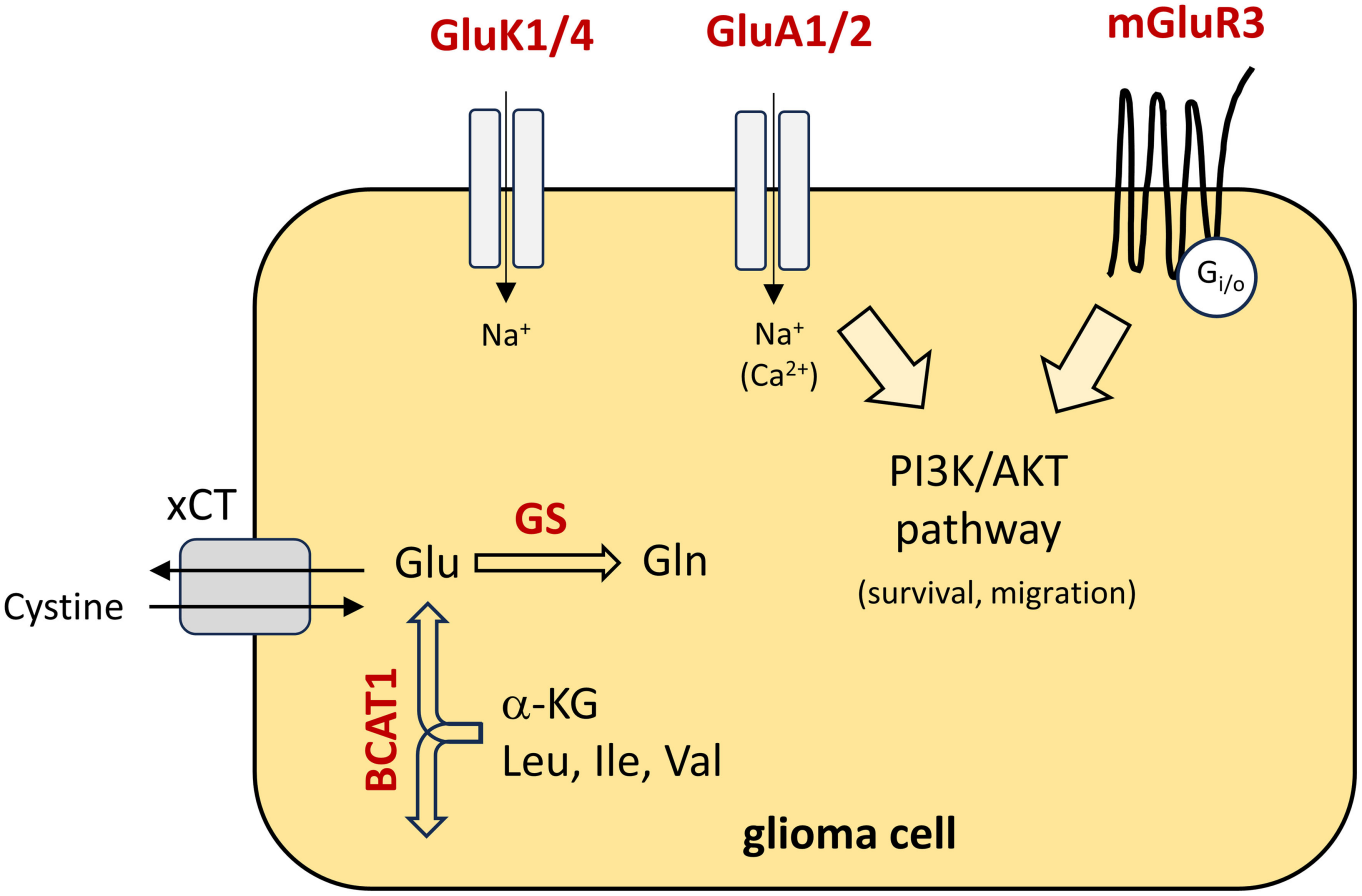
Schematic presentation of the pathophysiological function of the biomarker panel in glioma cells. Biomarker candidates are highlighted in red colour and their functions are illustrated. Briefly, in the cytoplasm, glutamate (Glu) is synthesised from α-ketoglutarate (α-KG) and branched-chain essential amino acids by BCAT1. In glioblastoma, glutamate is primarily released via cystine/glutamate antiporter solute carrier family 7 member 11 (xCT). Cystine is an essential precursor for glutathione synthesis, to counteract oxidative stress in fast-growing tumours. In addition, glutamate is catalysed to glutamine (Gln) by glutamine synthetase (GS). With respect to glutamate receptors, the metabotropic receptor mGluR3 is coupled to downstream signalling pathways like the PI3K/AKT pathway and contribute to migration and survival of the tumour cells. In part, AMPA receptors (GluA1/2) may also contribute to an activation of downstream signalling pathways due to calcium influx. Little is known on the function of kainate receptors (GluK1/4). Leu, Leucine; Ile, isoleucine; Val, Valine.

The Youden indices were used as cut-points and ΔCt values were calculated. Since TATA-binding protein (TBP) was used as housekeeping gene, ΔCt values may vary from the proposed values employing different housekeeping genes or adapted real-time PCR conditions. In our analysis, the gene panel was used to predict the correct tumor entity in 88% of the cases. A reduction of genes may reduce the power of prediction. Quite the opposite, including one or more genes away from glutamate signaling may further strengthen the approach. To sum up the functions of our biomarker candidates, the pathophysiological interactions are illustrated in **Figure 5**. One may speculate that in the long-term our biomarker panel could be integrated after initial imaging of the brain, to help on the decision for a subsequent surgical resection of a high-grade glioma or whether a more conservative therapy should be pursued in case of a brain metastasis.

Tumor-associated epilepsy presents not only a serious impact on the quality of life, but with respect to a status epilepticus, it is a neurological emergency associated with a high mortality. Hence, administration of anticonvulsants for seizure-control is often indicated (68). Anticonvulsants are administered prior to surgery to prevent seizures while excising the tumor mass. Since glutamate-mediated signaling was identified to contribute to both, glioma progression and tumor-associated seizures, targeting glutamate receptors like AMPAR may kill two birds with one stone (10, 11). Remarkably, in our study, no differences of the expression pattern within the glioblastoma cohort with respect to the prevalence of epileptic seizures were found. Epilepsy was less frequent in older patients, which is in line with a previous study by Iuchi et al. (69), though other studies reported no significant difference in age (45, 46). Yuen et al., 2012 reported a correlation of intracellular glutamate levels and seizures (16), but based on our dataset we could not confirm an association of receptor/transporter expression and an epileptic phenotype as previously suggested (16, 70). Since glutamate-mediated signaling was identified to be crucial in tumor growth and invasion (71, 72), glutamate receptor-mediated signaling of tumor-surrounding neurons and astrocytes may be altered.

Why could our expression pattern not distinguish between patients suffering from seizures and those without epilepsy? There could be at least three possibilities. First, the sample size of our study could be underpowered to identify genes associated with seizures. Second, functional pathologies like the disruption of the blood-brain barrier or perturbations of GABAergic neurotransmission may contribute to generation of seizures independent of gene expression (73). Furthermore, an increase in intracranial pressure due to bulk expansion and incidence of brain oedema may also provoke tumor-associated seizures. Those mechanisms may have masked our findings with respect to differential gene expression patterns.

One limitation in our study is the overall sample size. While brain metastases overall are not infrequently diagnosed, the treatment of the primary tumor is often clinically more urgent. As a result, the cranial tumor bulk is closely monitored but only in some cases excised. Obviously, access to a large database of metastases is limited. As we aimed for a feasible approach suitable for everyday use to differentiate high-grade glioma and brain metastasis, our analysis is based on tumor tissue samples expression, but not on single-cells data. Therefore, various cells other than cancer cells such as endothelial cells could also be subjected to expression analysis. One may speculate that single-cell analysis would reveal even more differences in the glutamatergic gene expression pattern.

## 5 Conclusion

To sum up, we identified seven genes (*GRIA1*, *GRIA2*, *GRIK1*, *GRIK4*, *GRM3*, *GLUL*, *BCAT1*) whose mRNA expression may serve as potential molecular biomarker candidates to distinguish glioblastoma and brain metastases tissue derived from surgical resections. The expression pattern could be of use to support the pathological assessment of the material taken from surgery without further resection volumes. Since a potential limitation of our study is the overall sample size, we encourage other groups to test these candidates to evaluate our proposed panel of genes. Especially, with respect to the xCT expression (*SLC7A11*), further investigations may reveal a higher correlation of the gene expression with glioblastoma.

## Data availability statement

The data presented in this study are available on reasonable request from the corresponding author.

## Ethics statement

The studies involving humans were approved by Ethics committee of the University Medical Center Rostock St.-Georg-Str. 108 18055 Rostock. The studies were conducted in accordance with the local legislation and institutional requirements. The participants provided their written informed consent to participate in this study.

## Author contributions

FL: Data curation, Writing – original draft, Conceptualization, Formal analysis, Visualization. RG: Data curation, Investigation, Visualization, Writing – review & editing. AE: Investigation, Writing – review & editing. KP: Investigation, Writing – review & editing. GR: Data curation, Visualization, Writing – review & editing. CM: Resources, Writing – review & editing. BS: Resources, Writing – review & editing. CH: Resources, Writing – review & editing. DD: Resources, Writing – review & editing. ML: Resources, Writing – review & editing. RK: Writing – review & editing. TF: Resources, Writing – review & editing. TK: Conceptualization, Writing – review & editing.
