# Supplementary material for: A glutamatergic biomarker panel enables differentiating Grade 4 gliomas/astrocytomas from brain metastases
Source: Front Oncol. 2024 May 21;14:1335401. doi: 10.3389/fonc.2024.1335401 (PMC11148222; doi:10.3389/fonc.2024.1335401)
Supplement: Supplementary file 1 [file DataSheet_1.pdf]

Supplementary Material

Supplementary Table 1: Grade 4 glioma/astrocytoma cohort

Patient ID	Gender	Age at diagnosis (year)	Location	Epilepsy	IDH1 status	MGMT status
HHG_01	f	69	left frontal	no	WT	methylated
HHG_02	m	68	left frontal	yes	WT	methylated
HHG_03	m	80	left parietal	no	WT	unmethylated
HGG_04	m	29	right frontal	yes	WT	unmethylated
HGG_05	m	53	right temporal	no	WT	methylated
HGG_06	m	63	left temporoparietal	no	WT	unmethylated
HGG_07	m	39	left parietal	no	R132H	unmethylated
HGG_08	m	62	left parietooccipital	yes	WT	unmethylated
HGG_09	m	50	left temporal	yes	WT	unmethylated
HGG_10	f	65	right multifocal	no	WT	unmethylated
HGG_11	f	54	right temporal	yes	WT	methylated
HGG_12	f	91	right temporal	yes	WT	unmethylated
HGG_13	m	62	right parietal	yes	WT	unmethylated
HGG_14	m	70	right frontal	yes	WT	methylated
HGG_15	f	76	right frontal	no	WT	unmethylated
HGG_16	f	77	right frontal	no	WT	methylated
HGG_17	f	79	bifrontal	no	WT	methylated
HGG_18	f	66	left temporal	yes	WT	unmethylated
HGG_19	m	44	right parietooccipital	yes	R132H	methylated
HGG_20	m	84	right temporal	yes	WT	unmethylated
HGG_21	f	43	right frontal	yes	WT	methylated
HGG_22	m	73	left temporal	no	WT	unmethylated
HGG_23	m	71	right frontotemporal	no	WT	methylated
HGG_24	f	47	left parietal	yes	WT	unmethylated
HGG_25	m	79	right frontal	no	WT	unkown
HGG_26	f	72	left frontal	no	WT	methylated
HGG_27	f	69	right temporal	unkown	WT	unmethylated
HGG_28	m	40	left parietal	yes	WT	unmethylated
HGG_29	m	49	right frontal	yes	WT	methylated
HGG_30	m	50	left frontal	yes	WT	unmethylated
HGG_31	f	72	right frontal	no	WT	unmethylated
HGG_32	m	79	left temporal	yes	WT	unmethylated
HGG_33	m	73	right temporal	no	WT	methylated
HGG_34	m	43	right temporooccipital	yes	WT	unmethylated
HGG_35	f	72	right temporo-parieto-occipital	no	WT	methylated

Supplementary Table 2: Brain metastasis cohort

**Patient ID**	**Gender**	**Age at diagnosis (year)**	**Location**	**Epilepsy**	**Primary tumor**
MET_01	f	66	right parietal, left temporal	yes	lung cancer (NSCLC)
MET_02	f	60	left frontal	no	colorectal cancer
MET_03	f	49	right frontal	yes	colorectal cancer
MET_04	f	43	left cerebellar	no	breast cancer[a]
MET_05	f	49	left frontal	yes	melanoma
MET_06	f	54	right cerebellar	yes	lung cancer (SCLC)
MET_07	f	75	left occipital	no	cervical cancer
MET_08	m	71	left temporal, right occipital	no	lung cancer (NSCLC)
MET_09	f	67	left frontotemporal	no	lung cancer (NSCLC)
MET_10	m	58	right cerebellar	no	lung cancer (NSCLC)
MET_11	f	59	right occipital	no	lung cancer (SCLC)
MET_12	m	58	right cerebellar	no	lung cancer (NSCLC)
MET_13	f	66	left cerebellar	no	breast cancer[a]
MET_14	f	49	cerebellar	no	lung cancer (NSCLC)
MET_15	m	52	cerebellar	no	lung cancer[a]
MET_16	m	n.a.	corcial	N/A	lung cancer[a]
MET_17	m	n.a.	right occipital	no	lung cancer[a]
MET_18	m	75	right frontal	no	rectal cancer
MET_19	f	42	left cerebellar	no	breast cancer[a]
MET_20	m	n.a.	occipital	N/A	renal cell carcinoma[a]

n.a. : not accessible
[a] : not specified further

Supplementary Table 3: Primers

Gene	Forward (5´-3´)	Reverse (5´-3´)
GRIN1	AGGAACCCCTCGGACAAGTT	CTCTCCAGTCGTCACCAGGT
GRIN2A	TGGACGTGAACGTGGTAGC	CCCCCATGAATGCCCAAGAT
GRIN2B	TTCCGTAATGCTCAACATCATGG	TGCTGCGGATCTTGTTTACAAA
GRIN2C	ACCTGCCATTTTGCTGGGG	GGCTACACTGCTGATCTCGT
GRIN2D	CTGGCCTCACTGGATCTGG	GGAAGGAAACCATAATCACGCA
GRIN3A	GACGCCCTCCTATTTGCCG	CCACGGTATGGCACACACT
GRIN3B	TGTCTCGGCACTTTAAGGTGT	GGTGTTCCAACAGCGTTACCA
GRIA1	GGTCTGCCCTGAGAAATCCAG	CTCGCCCTTGTCGTACCAC
GRIA2	TGTTGGAGTCCACGATGAACG	GCAAGATTTACTGGGGTTCCTAA
GRIA3	CGAGAGGGGTGTATGCCATC	GAAGCTAGGCGTAACAAAGGAT
GRIA4	ATTGGTGTCAGCGTGGTCTTA	CCAGGGAAAACCAGAGGCT
GRIK1	ACTCAGGATCGGAGGGATTTT	GGTGACTGCAAACTTGAAAGC
GRIK2	AGCGTCGGTTAAAACATAAGCC	GTTTCTTTACCTGGCAACCTTCT
GRIK3	AGAGAGCAGCGTTCCTTCTG	CGGCGGTCATTGAATGTGT
GRIK4	TGAGGATCGCTGCTATCTTGG	CGTACTCGCTGTCTCTGAGAA
GRIK5	GATCAACGGGATCATCGAGGT	GTGTCCGTGGTCTCGTACTG
GRM1	CAGCCGATTCGCTTTAGCC	GGGATCGCGGTACTGAAGTTG
GRM2	CTATGGCGAGACAGGCATTGA	CATCCTCAGAACGGGTGAACA
GRM3	GCACCTCAACAGGTTCAGTGT	TGGTGGAGTCGAGGACTTCC
GRM4	GACAACAGCCGCTACGACTT	GAGGCCACTGTGGACACAT
GRM5	AATCTCCCGATGTCAAGTGGT	AGGGTTTCGGTGGTTTGTTTC
GRM6	CCACACAGCGTGATTGACTAT	GCAGCCGATGAGAGACAGAT
GRM7	GGCTGGAAGCGATGCTCTAC	TGTTCGAGCGCGTAAGTGTC
GRM8	CCAGAGCTAAGTGATAACACCAG	TCTGTGACTGAGCAATGCAAA
SLC1A2	CAGACTAGTGCTCCTGCGAT	CCTCTTAAGCCCCGTCAAGG
GLUL	TCATCTTGCATCGTGTGTGTG	CTTCAGACCATTCTCCTCCGG
BCAT1	CAACTATGGAGAATGGTCCTAAGCT	TGTCCAGTCGCTCTCTTCTCTTC
IDH1	CGGTCTTCAGAGAAGCCATT	GCAAAATCACATTATTGCCAAC
SLC7A11	TGCTGGGCTGATTTATCTTCG	GAAAGGGCAACCATGAAGAGG
GAPDH	CCACTCCTCCACCTTTGAC	ACCCTGTTGCTGTAGCCA
TBP	TCGGAGAGTTCTGGGATTGT	CACGAAGTGCAATGGTCTTT

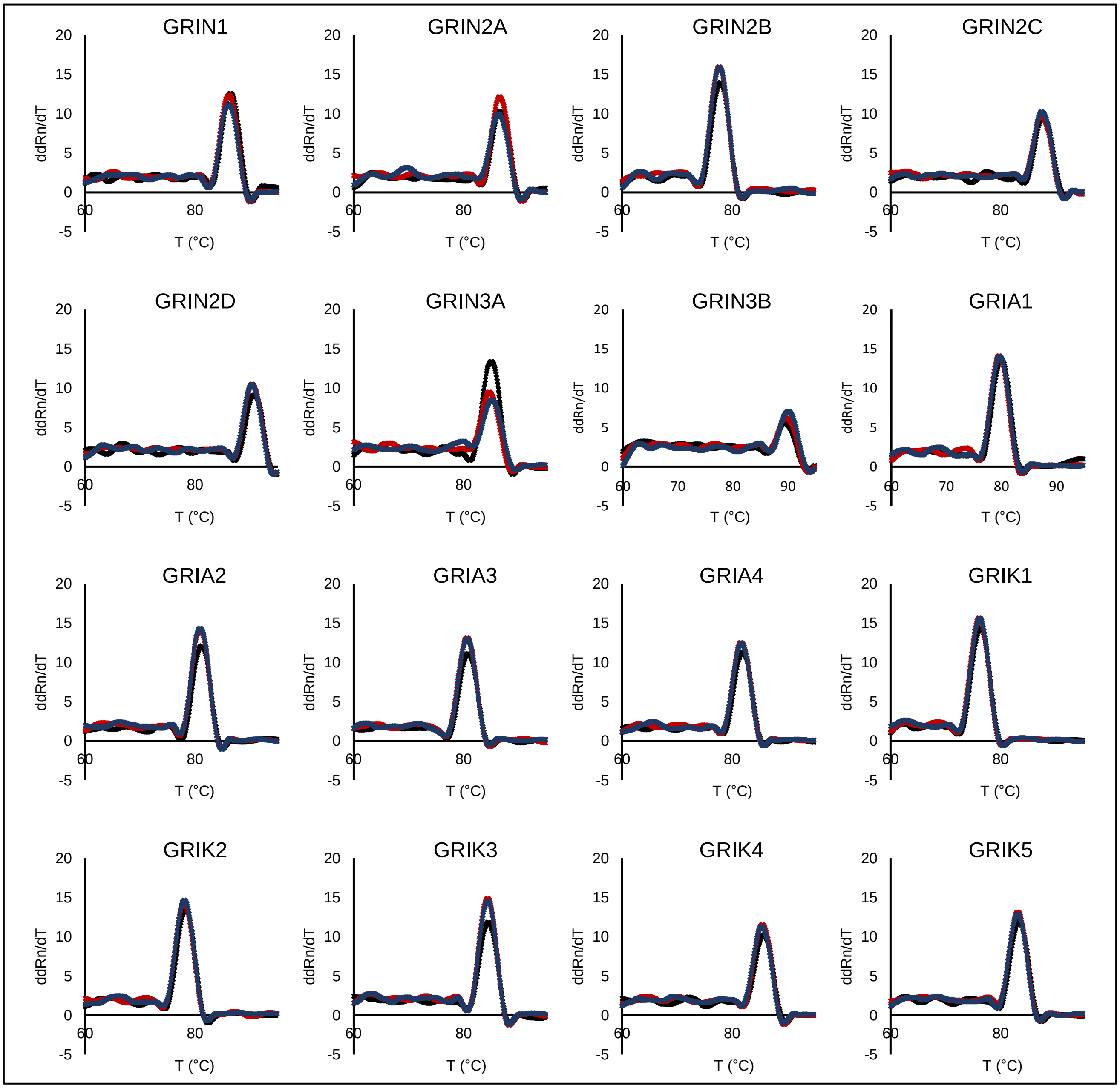

Supplementary Figure 1: Sample traces of melting curves of ionotropic glutamate receptors. Data represent relative fluorescence signal (ddRn/dT) for three different glioblastoma samples (HGG_33: black, HGG_34: red, HGG_35: blue) of primer pairs to specifically analyze expression of ionotropic glutamate receptors. Temperature was increased in steps of 0.1 °C from 60°C to 95°C.

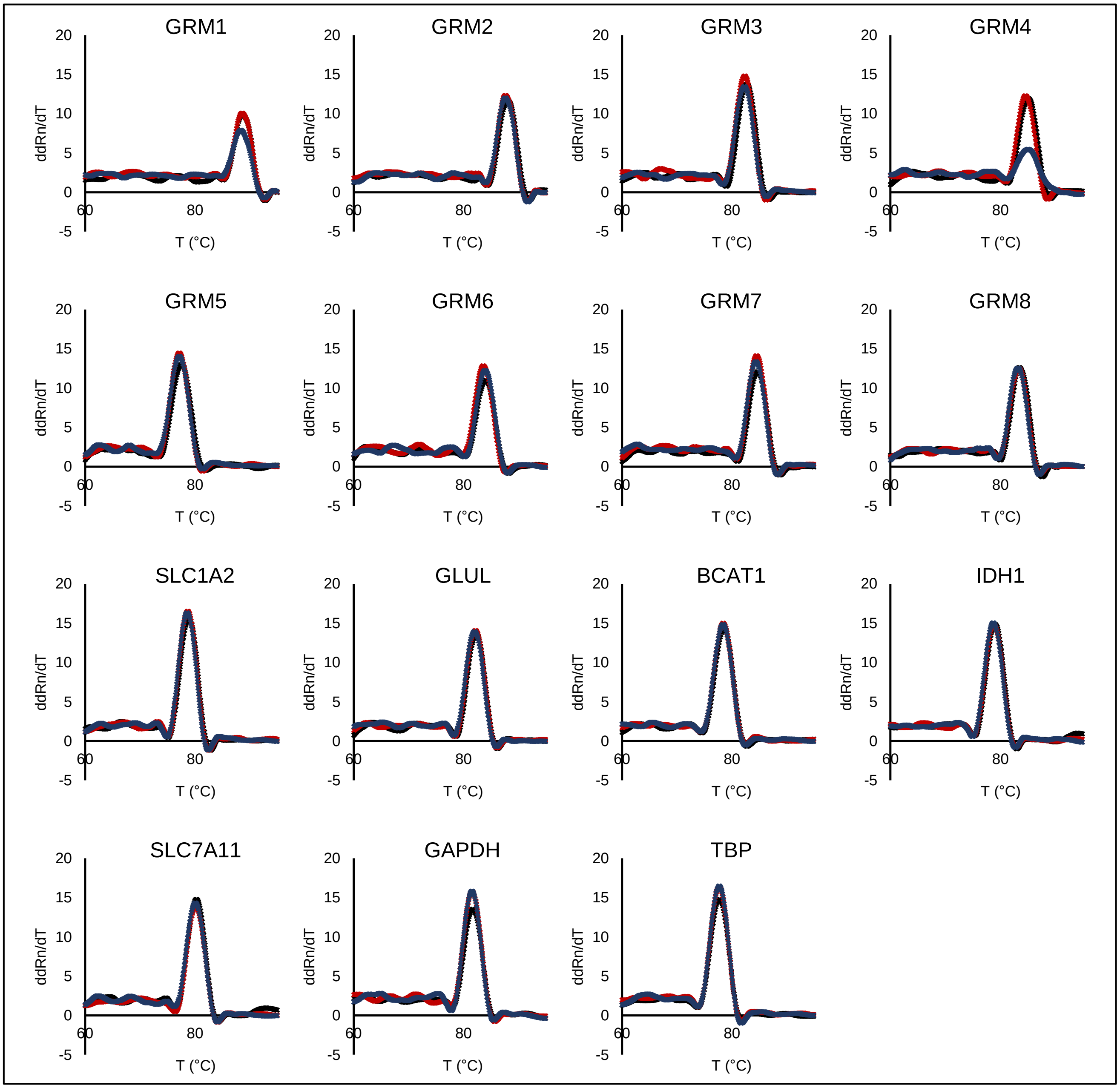

Supplementary Figure 2: RT-PCR melting curves of primers for metabotropic glutamate receptors and genes associated with glutamate shuttling & metabolism. Data represent relative fluorescence signal (ddRn/dT) for three different glioblastoma samples (HGG_33: black, HGG_34: red, HGG_35: blue) of primer pairs to specifically analyze expression of metabotropic glutamate receptors and genes associated with glutamate shuttling & metabolism. Temperature was increased in steps of 0.1 °C from 60°C to 95°C.

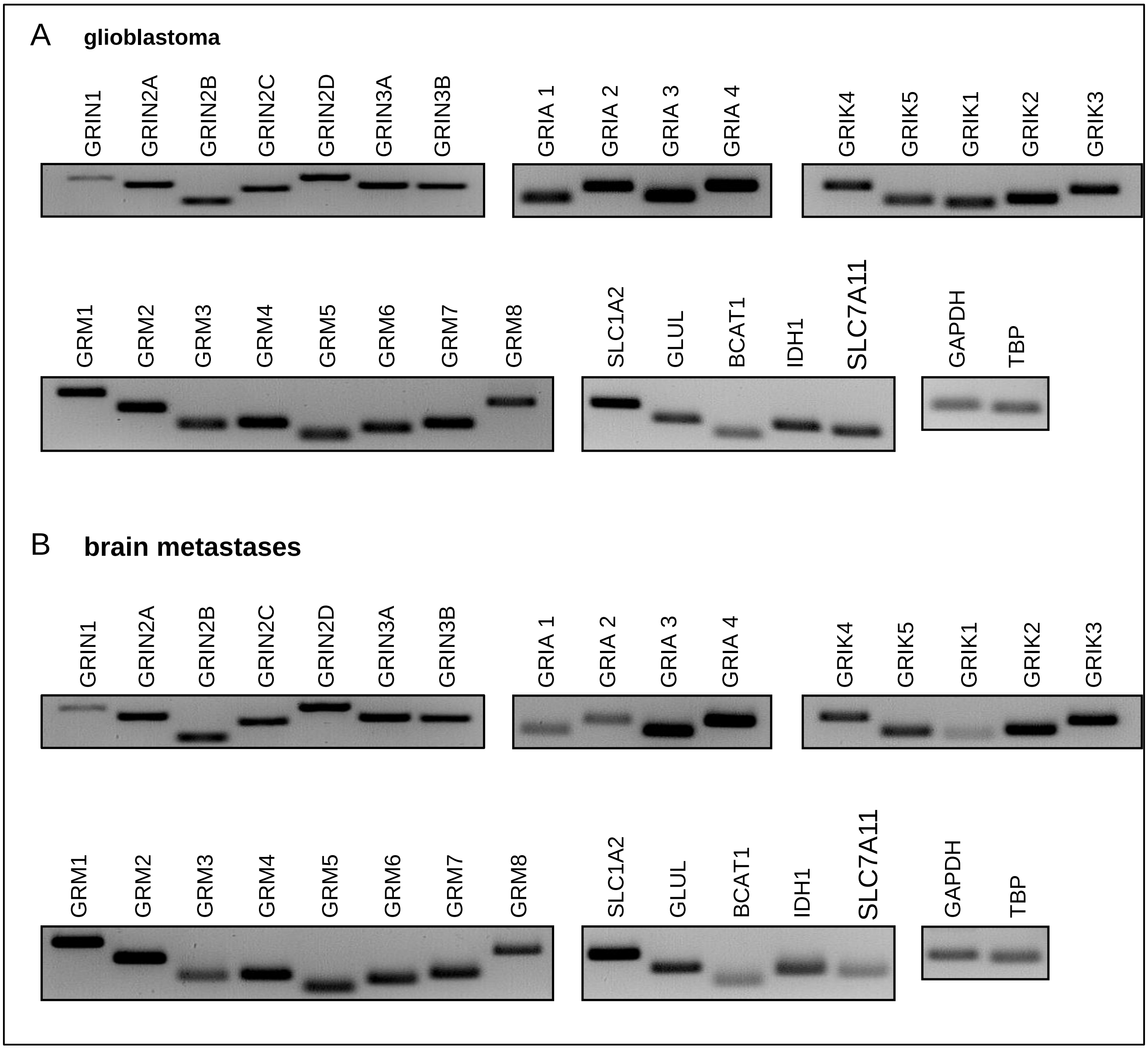

Supplementary Figure 3: Visualization of PCR products by gel electrophoresis of (**A**) glioblastoma and (**B**) brain metastases tissue samples. Following amplification in the RT-PCR (see Materials and methods for details), products were conducted to gel electrophoresis. For both tumor entities, only one PCR product was detected for each gene examined.

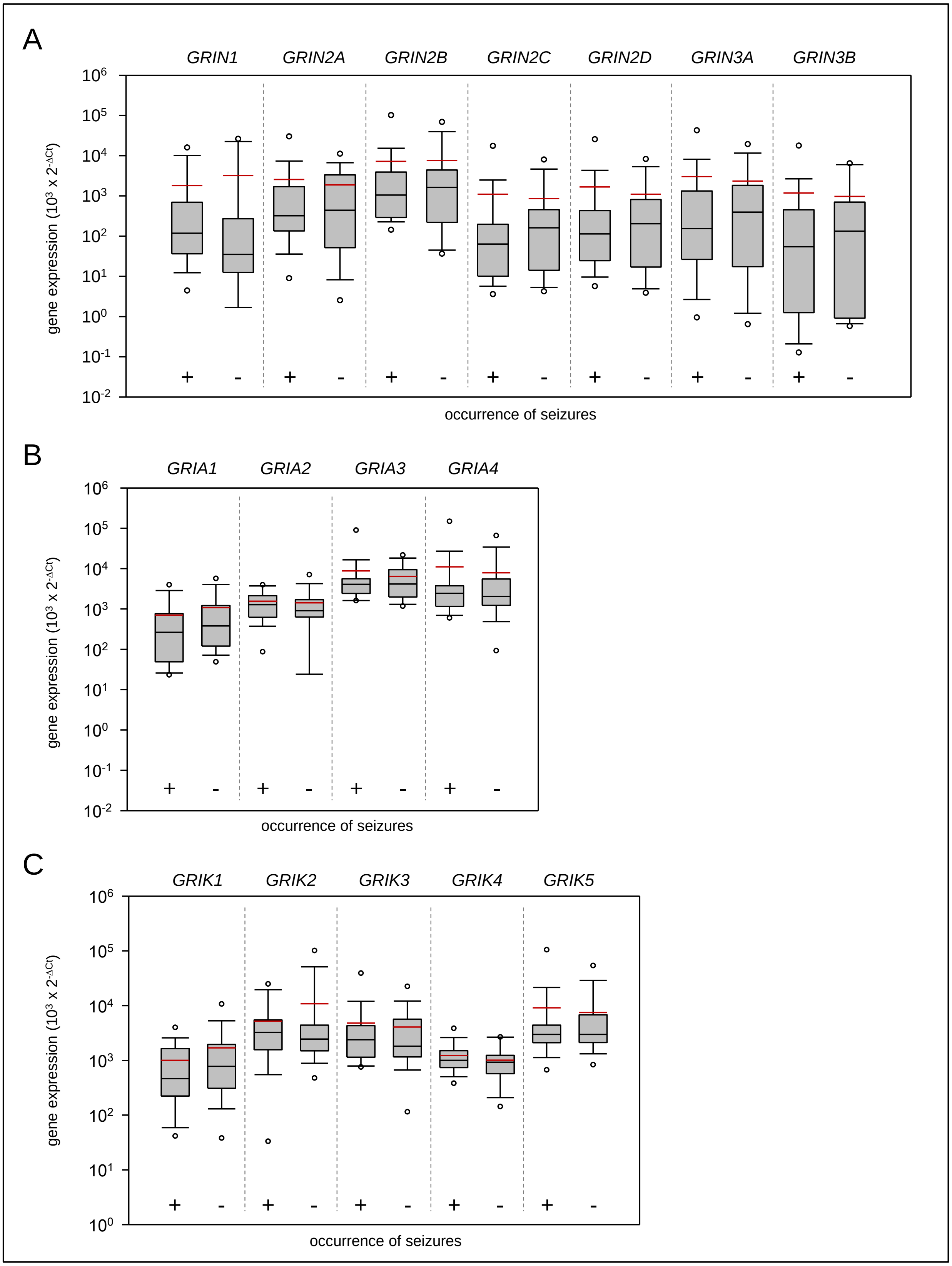

Supplementary Figure 4: Expression of ionotropic glutamate receptors in grade 4 glioma and astrocytoma. No differences in gene expressions were found in the comparison of cohorts w/ (n=18) and w/o (n=16) known epilepsy of the patients (Mann-Whitney U test).

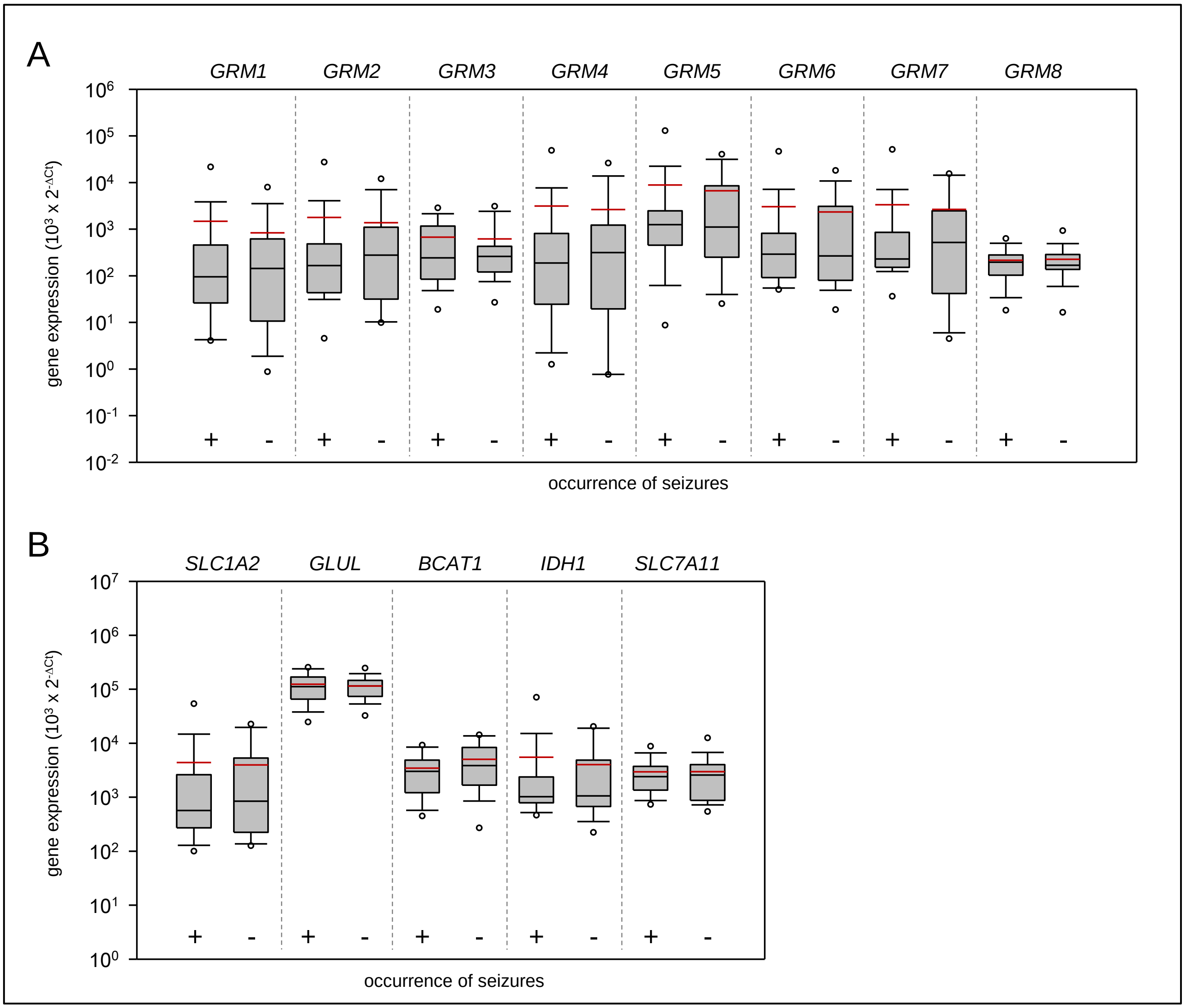

Supplementary Figure 5: Expression of metabotropic glutamate receptors and key players of glutamate homeostasis in grade 4 glioma and astrocytoma. No differences in gene expressions were found in the comparison of cohorts w/ (n=18) and w/o (n=16) known epilepsy of the patients (Mann-Whitney U test).

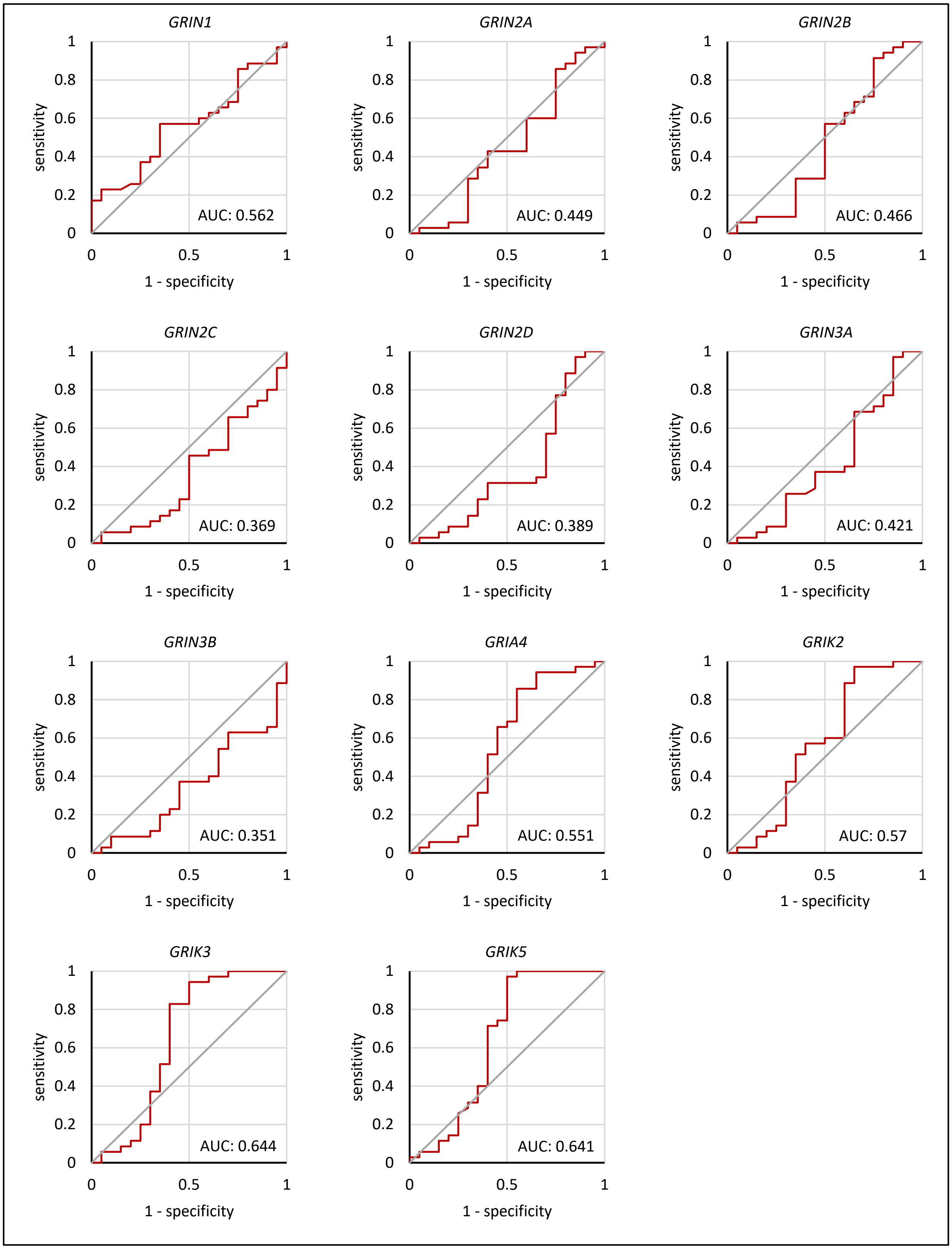

Supplementary Figure 6: ROC analysis of gene expression of ionotropic glutamate receptors. Please note that only genes with an AUC <0.8 are presented (for genes with AUC >0.8 see Figure 3).

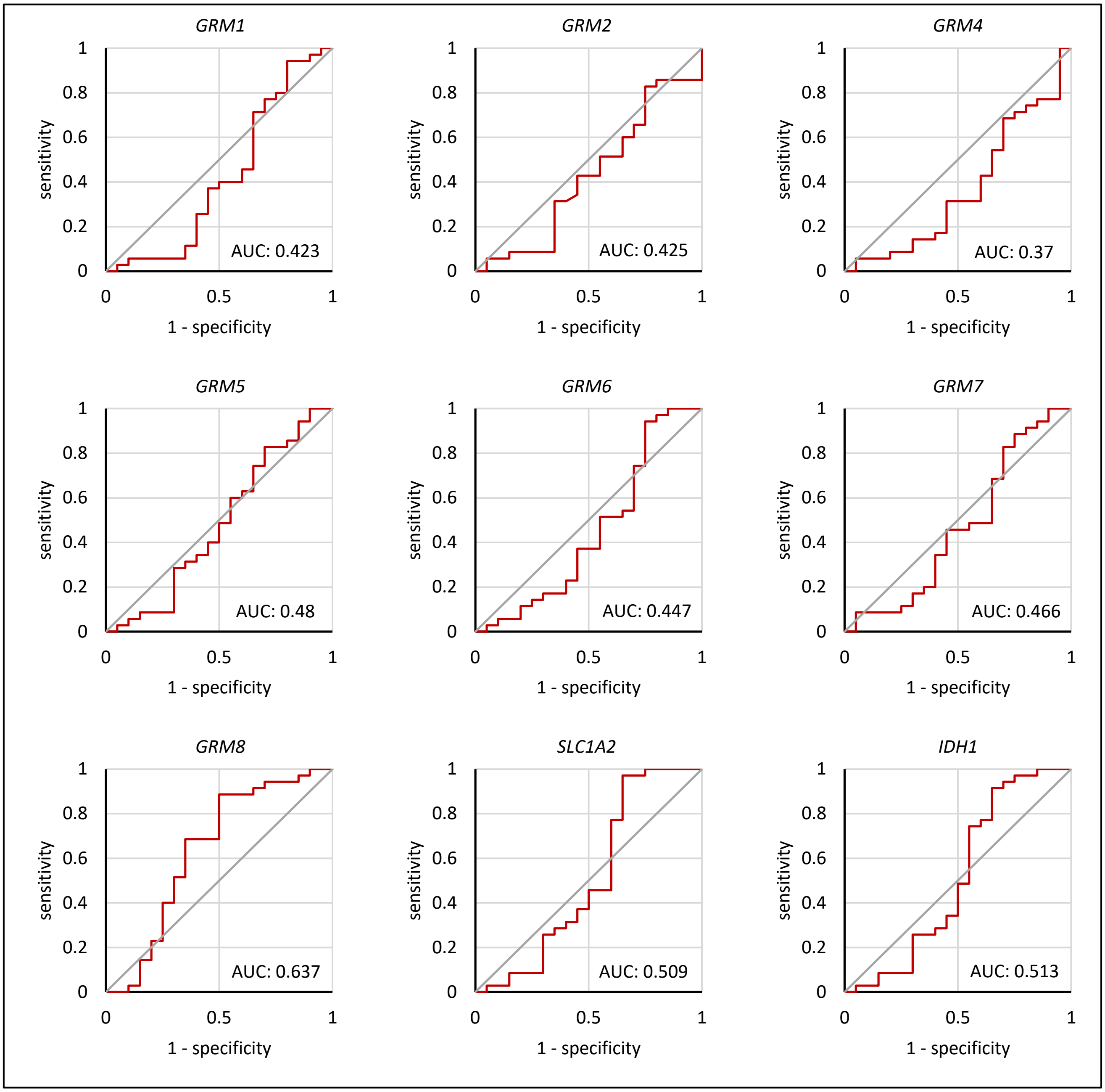

Supplementary Figure 7: ROC analysis of gene expression of metabotropic glutamate receptors and key players of glutamate homeostasis. Please note that only genes with an AUC <0.8 are presented (for genes with AUC >0.8 see Figure 3).
